# Properties of bacterial communities attached to artificial substrates in a hypereutrophic urban river

**DOI:** 10.1186/s13568-018-0545-z

**Published:** 2018-02-16

**Authors:** Xianlei Cai, Ling Yao, Qiyue Sheng, Luyao Jiang, Randy A. Dahlgren, Ting Wang

**Affiliations:** 1 Key Laboratory of Watershed Science and Health of Zhejiang Province, Wenzhou Medical University, Wenzhou, 325035 China; 2 Southern Zhejiang Water Research Institute, Wenzhou, 325035 China; 3 Department of Land, Air and Water Resources, University of California, Davis, CA 95616 USA

**Keywords:** Biofilms, High-throughput sequencing, Microbial communities, Urban rivers

## Abstract

Bacterial communities of biofilms growing on artificial substrates were examined at two time periods (7 and 14 days) and two locations (lentic and lotic areas) in a hypereutrophic urban river of eastern China. Previous studies in this river network indicated that variations of microbial communities were the major factor affecting the distribution of antibiotic resistant genes highlighting the importance of understanding controls of microbial communities. Bacterial communities associated with biofilms were determined using epifluorescence microscopy and high-throughput sequencing. Results showed that sampling time and site had significant effects on the abundances of surface-associated bacteria. No significant differences were found in the number of surface-associated bacteria between two substrate types (filament vs. slide). Sequencing revealed microbial communities attached to artificial substrates in a hypereutrophic urban river were composed of 80,375 OTUs, and distributed in 47 phyla. *Proteobacteria* and *Cyanobacteria*/*Chloroplast* were the two dominant phyla, followed by *Planctomycetes*, *Actinobacteria*, *Verrucomicrobia*, *Firmicutes* and *Bacteroidetes*. Taxonomic composition showed ammonia-oxidizing microorganisms, fecal indicator bacteria and pathogens enriched in attached microbial communities, especially the ammonia-oxidizing *Nitrosomonas* bacteria. These results indicated that there were significant temporal and intra-river heterogeneity of attached microbial community structure, but no significant difference in community composition was detected between the two substrate types.

## Introduction

Rivers are an important resource for human society and ecosystems by providing water for consumption, agriculture, industry and replenishing other freshwater ecosystems (Araya et al. 2003; Vörösmarty et al. 2010). Compared to wildland rivers, rivers that runthrough urban centers are highly impacted by human activities, such as wastewater discharge, channelization, and bank reinforcement. Consequently, urban rivers tend to be among the most degraded aquatic ecosystems in the world. In China, rapid economic growth and urbanization within the past several decades has resulted in dramatically degraded water quality and decline in ecosystem function of urban rivers. About 80% of Chinese urban rivers were reported to be heavily polluted and degraded, especially in the densely populated eastern coastal plains (Cai et al. 2016; Qiu 2011; Zhang and Xu 2011). Hence, improved knowledge of the ecological characteristics of urban rivers in China is essential to assess local public health concerns and support river ecological restoration.

Aquatic biofilms colonize various surfaces in riverine systems and play very important roles in aquatic ecology (e.g., food web and habitat) and water quality dynamics (e.g., degradation and transformation). The surfaces of artificial or natural substrates immersed in the aquatic environments are rapidly colonized by microorganisms, primarily bacteria, forming many unique microbial ecosystems, such as epiphyton, epipelon, epixylon, epilithon, epipsammon, and so on (Azim et al. 2005; Li et al. 2014; Pohlon et al. 2010; Qian et al. 2007; Salta et al. 2013). The bacterial communities within these microecosystems are highly sensitive to environmental fluctuations (Araya et al. 2003; Paerl et al. 2003). They are also hot spots for the turnover of allochthonous organic matter and mineral nutrients (Augspurger et al. 2008; Geesey et al. 1978; Pohlon et al. 2010) including anthropogenic pollutants, particularly in hypereutrophic urban rivers lacking macroorganisms. In addition, the surface-associated microbial communities have been widely used in the remediation of contaminated waters (Gil-Allué et al. 2015; Wu et al. 2012, 2014). Artificial substrates have been widely used to collect and study surface-associated microorganisms (Brummer et al. 2000; Jones et al. 2007; Wang et al. 2013), such as in examining water treatment processes, because they are stable and easily obtained (Dong et al. 2003; Guzzon et al. 2008; Wan et al. 2016). Considering the important significance of surface-associated bacterial communities in urban rivers, especially for their possible applications in river restorations, there is a critical need to better understand the composition and functions of bacterial biofilm communities in urban rivers.

The identification of bacterial communities at higher taxonomic resolution is a powerful tool to better understand the intricacies of microbial ecological processes. Rapid advances in high-throughput sequencing enable low cost detection of bacterial communities at high-resolution, even rare taxa with low relative abundance (Lemos et al. 2011; Logares et al. 2012). A previous report from the river network examined in this study indicated that variations in microbial communities was the major factor affecting the distribution of antibiotic resistant genes highlighting the importance of understanding controls of microbial communities (Zhou et al. 2017). Therefore, in order to better understand surface-associated bacterial communities in hypereutrophic urban rivers, we utilized high-throughput sequencing to explore the bacterial community composition. The primary objective of this work was to investigate the properties of bacterial communities attached to artificial substrates in a hypereutrophic urban river for the purpose of enhancing river ecological restoration activities and assessing human health concerns.

## Materials and methods

### Study site and experimental design

**Fig. 1:**
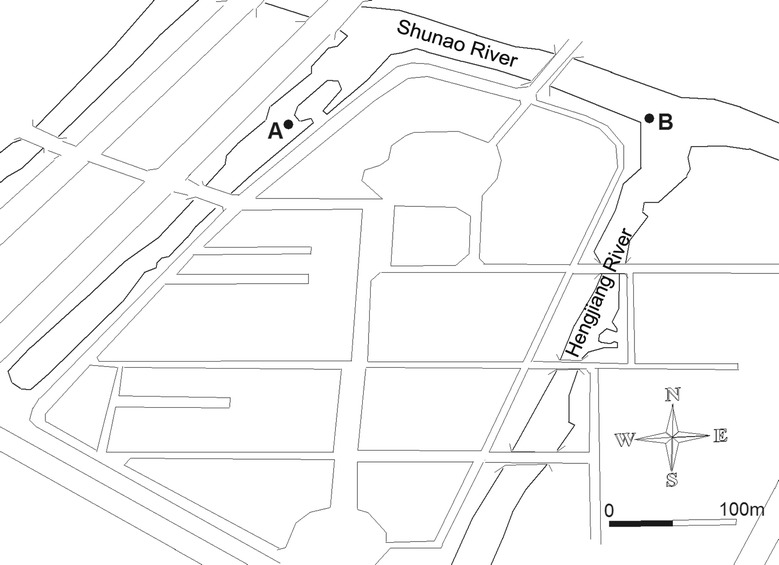
Location of the study sites in Shunao River and Hengjiang River tributaries of the Wen-Rui Tang River

The study was conducted in the Wen-Rui Tang River, a typical coastal plain river network, located in Wenzhou, eastern China. This river flows through a densely populated area (~ 3.0 million city and 9.1 million regional population) where garbage and untreated industrial/municipal wastes are often dumped indiscriminately into the river network. Due to rapid economic development and urbanization, water quality in the Wen-Rui Tang River has degraded dramatically (Lu et al. 2011; Yang et al. 2013). According to China surface water quality standards (GB3838-2002), Wen-Rui Tang River water quality is highly degraded, V grade or less, due to the high contents of total nitrogen, total phosphorus and ammonium (Mei et al. 2014). The Shunao and Hengjiang Rivers are tributaries of the Wen-Rui Tang River and their water quality is representative of the impacts resulting from rapid urbanization that has occurred over the past two decades. A lentic-dominated site along the Shunao River (Site A: 27.929854°N, 120.705249°E), and a lotic-dominated site located at the confluence of the Shunao and Hengjiang Rivers (Site B: 27.929687°N, 120.708203°E) were selected for investigation (Fig. 1). Dissolved oxygen (DO) was recorded at the time of water collection using a multi parameter probe (YSI 650MDS, YSI Incorporated, Yellow Springs, Ohio). Water samples were collected at the mid-point of the 14 day experiment for analysis of total nitrogen (TN), ammonium (NH_4_^+^–N), nitrate (NO_3_^−^–N), nitrite (NO_2_^−^–N), total phosphorus (TP), orthophosphate (PO_4_^3−^–P) and total organic carbon (TOC) were analyzed according to Jin and Tu (1990).

Polyethylene slides are widely used for the study of biofilms in natural aquatic environments while polyethylene filaments are a standard material used in biofilm studies for water treatment technologies. Therefore, we deployed both of these substrates in this study to examine the development of bacterial communities under hypereutrophic conditions in this urban river network. The substrate materials were thoroughly washed with tap water, rinsed with distilled water, and then air dried before deployment. After measuring their surface area, these materials (slide: 5 × 1.5 × 0.16 cm; filaments: length 10 cm, diameter 0.06 cm, 9 filaments per replicate) were anchored at a 30-cm depth below the water surface. Artificial substrates were harvested 7 and 14 days after deployment in April 2016. Triplicate artificial substrates were randomly collected at each time point. Collected samples were placed in sterile bags and transported to the laboratory (~ 0.5 h) for immediate processing.

Biofilms attached to artificial substrates were detached with a sterile soft toothbrush in 400 mL sterile distilled water, and then treated with probe sonication as follows: ultrasonic time 3 s, interval time 8 s, repetition 75 times, power 600 W (Cai et al. 2013, 2014). After detachment, 10 mL of sample was immediately fixed with 2% formaldehyde (final concentration) and cooled at 4 °C for later direct counting of cells. Simultaneously, 150 mL of sample was filtered onto a 0.2 μm polycarbonate membrane filter (47 mm diameter, Millipore), and stored at − 20 °C for subsequent molecular analyses.

### Cell counts

The number of bacterial cells was determined microscopically after staining with DAPI (4′,6-diamidino-2-phenylindole; Porter and Feig 1980). DAPI was added to the samples at a final concentration of 1 μg/mL, and samples were allowed to incubate at room temperature for 10 min. Then samples were filtered onto black polycarbonate filters (0.2 μm pore size, 25 mm diameter; Millipore) with a < 10 mmHg vacuum. Cells were enumerated using a Leica fluorescent microscope (DM4000B, Germany). A minimum of 20 randomly selected fields of view were counted per sample.

### DNA extraction, 16S rDNA amplification and sequencing

Total DNA was extracted from frozen filters with the E.Z.N.A.^®^ Water DNA Kit (Omega, USA) according to manufacturer’s protocols. The region-specific bacterial/archaeal primer pairs for DNA amplification were S-D-Bact-0341-b-S-17, 5′-CCTACGGGNGGCWGCAG-3′, and S-D-Bact-0785-a-A-21, 5′-GACTACHVGGGTATCTAATCC-3′ (Klindworth et al. 2013), with Illumina adapters added. PCR reactions were performed using KAPA HiFi HotStart ReadyMix PCR Kit (2×) (Kapa Biosystems, US) in a 25 μL reaction volume with an initial denaturation step at 95 °C for 3 min, followed by 25 cycles each of denaturation at 95 °C for 30 s, annealing at 55 °C for 30 s, and extension at 72 °C for 30 s, followed by a final extension at 72 °C for 5 min. Sequencing was performed on the Illumina platform at Shanghai Xiangyin Biotechnology Co., Ltd. Denoised sequences were aligned and sorted into operational taxonomic units (OTUs) at the 97% similarity level, which corresponds approximately to the species level. Taxonomy was assigned using the Ribosomal Database Project (RDP) classifier (Cole et al. 2014). Good’s coverage, abundance-based coverage estimator (ACE), Chao1 richness estimator, Shannon index, and Simpson index were calculated based on the OTU data. All sequence data from this study were submitted to the National Center for Biotechnology Information (NCBI) Sequence Read Archive (SRA) under Accession Number SRP119548.

### Statistical analysis

Univariate data were expressed as means and standard errors, and considered statistically significant at p values < 0.05. Multivariate statistical analysis was carried out using Canoco for Windows 5.0 (Ter Braak and Šmilauer 2012) and PAST (PAleontological STatistics v1.81) (Hammer et al. 2001). Multivariate analyses of community structure were carried out on data for genus relative abundance. Principal component analysis (PCA) was used to investigate how community composition varied between sites, artificial substrates, and sampling times. Analysis of similarity (ANOSIM) was used to statistically test the effects of sampling time, substrate type, and site on bacterial community structure. ANOSIM is a nonparametric method to test for differences between two or more groups, based on any distance measure (Clarke 1993). In the present study, the distance indices of genus relative abundance were calculated using Bray–Curtis indices. The significance was computed by permutation of group membership, with 10,000 replicates. ANOSIM generates a test statistic, R, and the magnitude of R is indicative of the degree of separation between groups, with a score of 1 indicating complete separation, and 0 indicating no separation.

## Results

### Water quality characterization

The physicochemical water quality characteristics at the two study sites in the Wen-Rui Tang River are displayed in Table 1. Both sites had high nitrogen, phosphorus, and total organic carbon concentrations. Dissolved inorganic nitrogen (NH_4_^+^–N and NO_3_^−^–N) was the main form of nitrogen while particulate forms of P were higher than dissolved PO_4_^3−^. Total phosphorus concentration was higher at Site B (lotic) compared to Site A (lentic), although both sites had similar orthophosphate concentrations. The higher energy lotic conditions of Site B may enhance resuspension of sediment particles along with their associated P fraction. Total organic carbon, a possible energy source for the microbial community, was similar between the two sites. Dissolved oxygen concentrations (4.3–5.5 mg/L) were considerable below saturation levels of ~ 9.0 mg/L reflective of an aquatic system with high concentrations of oxygen demanding substances (e.g., organic matter, NH_4_^+^–N).

**Table 1:** Physicochemical analysis of surface waters at the two study sites in April 2016

Parameter	Site A (lentic)	Site B (lotic)
DO (mg/L)	4.34	5.51
TN (mg/L)	3.62	4.55
NH_4_^+^–N (mg/L)	2.35	1.84
NO_3_^−^–N (mg/L)	1.19	2.39
NO_2_^−^–N (mg/L)	0.06	0.10
TP (mg/L)	0.30	1.29
PO_4_^3−^–P (mg/L)	0.05	0.05
TOC (mg/L)	5.9	5.4

DO, dissolved oxygen; TN, total nitrogen; NH_4_^+^–N, ammonium; NO_3_^−^–N, nitrate; NO_2_^−^–N, nitrite; TP, total phosphorus; PO_4_^3−^–P, orthophosphate; TOC, total organic carbon

### Bacterial abundance

**Fig. 2:**
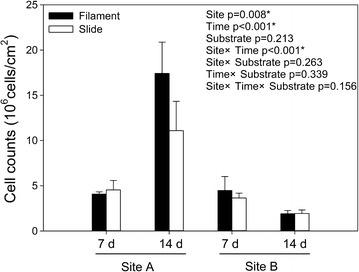
The effects of sampling time, Site (A = lentic, B = lotic) and substrate type on the number of surface-associated bacteria

The effects of sampling time (7 vs. 14 days), site (lentic vs. lotic) and substrate type (slide vs. filament) on the number of surface-associated bacteria are shown in Fig. 2. Both sampling time and site had significant effects on the number of surface-associated bacteria and there was also a significant interaction between sampling time and site. At Site A (lentic), the number of surface-associated bacteria showed a marked increase with time, especially for the filament substrate. In contrast, the number of surface-associated bacteria at Site B (lotic) slightly declined with time. No significant difference was found in the number of surface-associated bacteria between the two substrate types, but the number of bacteria attached to the filament was markedly higher than that attached to the slide on day 14 at Site A.

### Community composition, species richness and diversity

A number of microbial diversity indices for the 16S rDNA Illumina reads are shown in Table 2. The mean Good’s coverage ranged from 92.4 to 95.8%, indicating that the vast majority of phylotypes were detected. Results from high-throughput sequencing showed that microbial assemblages colonizing the artificial substrates were species-rich (maximum mean 9165 OTUs). In general, there were more OTUs on the filament than slide. Species richness and diversity of the surface-attached communities decreased from 7 to day 14.

**Table 2:** Estimates of richness and diversity for bacterial communities attached to different artificial substrates

Site	Type	Time (days)	Reads	Coverage (%)	OTUs	ACE	Chao1	Shannon
A (lentic)	Filament	7	69,778 ± 4579	92.4 ± 0.5	8004 ± 834	44,019 ± 8347	24,896 ± 3573	6.17 ± 0.16
		14	82,027 ± 976	95.0 ± 0.1	6106 ± 152	37,101 ± 3919	20,022 ± 1174	5.27 ± 0.45
	Slide	7	70,641 ± 2268	93.5 ± 0.1	6822 ± 331	38,532 ± 1559	21,785 ± 1238	5.58 ± 0.16
		14	95,310 ± 7856	95.8 ± 0.4	6039 ± 223	33,240 ± 1198	18,737 ± 247	5.48 ± 0.24
B (lotic)	Filament	7	97,206 ± 10,591	93.7 ± 0.6	9165 ± 657	46,751 ± 1789	27,021 ± 599	5.94 ± 0.40
		14	94,369 ± 5038	95.7 ± 0.2	6208 ± 809	33,298 ± 1436	18,665 ± 1047	4.45 ± 0.43
	Slide	7	88,072 ± 10,100	95.0 ± 0.6	6676 ± 1446	37,249 ± 5566	20,869 ± 3075	5.19 ± 0.47
		14	62,271 ± 16,122	94.0 ± 1.2	5071 ± 354	26,826 ± 3597	15,182 ± 1629	5.15 ± 0.48

ACE, abundance-based coverage estimator

**Fig. 3:**
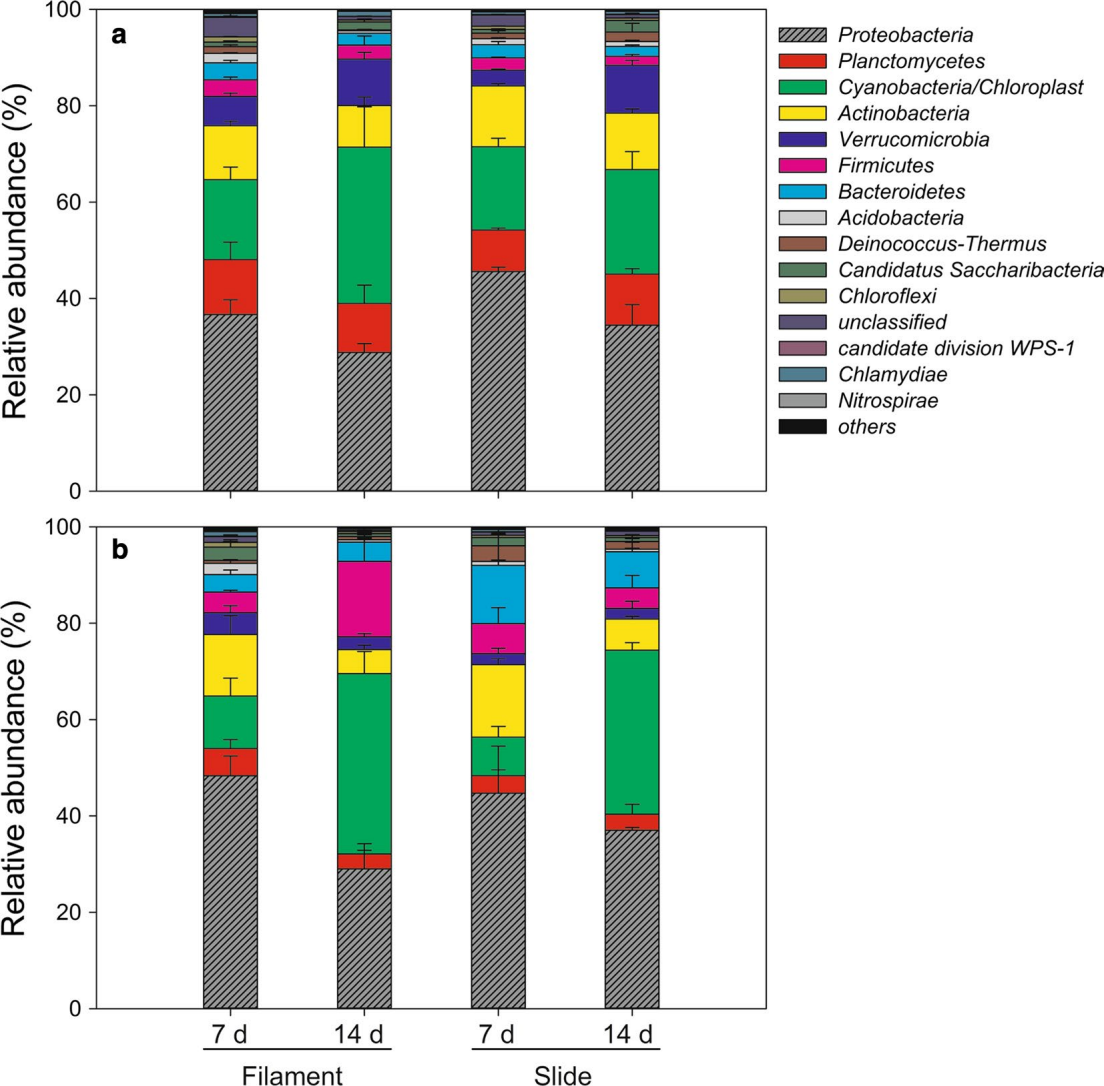
Effects of sampling time, site and substrate type on the dominant phyla of surface-associated bacteria. The sum relative abundance of dominant phyla exceeded 99%. **a** Site A = lentic; **b** Site B = lotic

Across all samples, 80,375 OTUs were identified from the data of high-throughput sequencing, and distributed among 47 phyla. The average relative abundance of dominant phyla (sum of the values > 99%) are shown in Fig. 3. The two predominant phyla were *Proteobacteria* and *Cyanobacteria*/*Chloroplast*; the sum of their abundance exceeded 50%. There were marked temporal variations in the relative abundance of dominant phyla. For example, the relative abundance of *Proteobacteria* was higher on day 7 than on day 14, whereas the relative abundance of *Cyanobacteria*/*Chloroplast* was lower on day 7 than day 14.

**Fig. 4:**
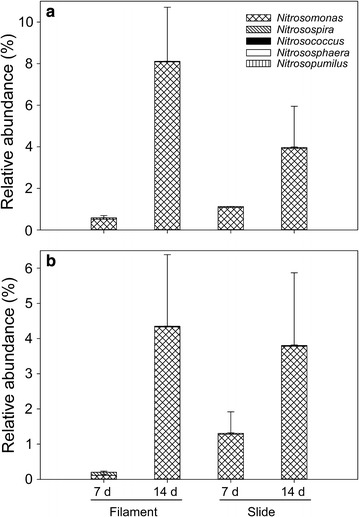
The effects of sampling time, site and substrate type on ammonia-oxidizing microorganisms in the community attached to artificial substrates. **a** Site A = lentic; **b** Site B = lotic

**Fig. 5:**
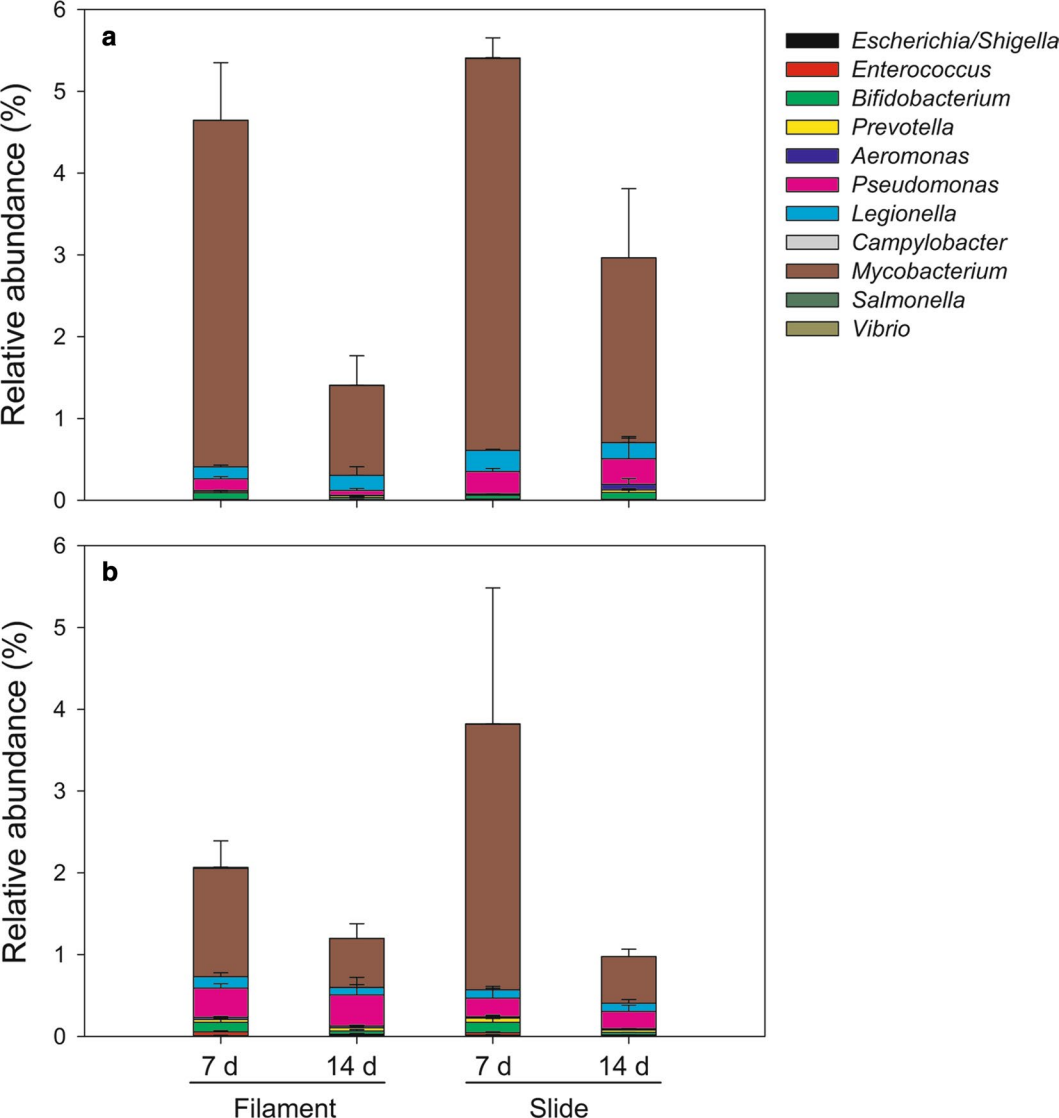
The effects of sampling time, site and substrate type on the genera of fecal indicator bacteria and pathogens in microbial community attached to artificial substrates. **a** Site A = lentic; **b** Site B = lotic

Due to the high ammonia nitrogen concentrations associated with the studied river system, the microbial community related with ammonia transformations is of great interest. Therefore, we especially focused on the ammonia-oxidizing microorganisms in the community attached to artificial substrates. *Nitrosomonas* was the predominant genus among ammonia-oxidizers identified (Fig. 4). The temporal variation in the sum of ammonia-oxidizer abundance showed an increasing trend with time, regardless of the attachment substrate. Additionally, considering that domestic sewage pollution is an important contributor to degraded water quality in the Wen-Rui Tang River, we focused on the genera of fecal indicator bacteria and pathogens. There were many fecal indicator bacteria and pathogens in the microbial community attached to artificial substrates (Fig. 5). The relative abundance of fecal indicator bacteria and pathogens decreased gradually with time.

**Fig. 6:**
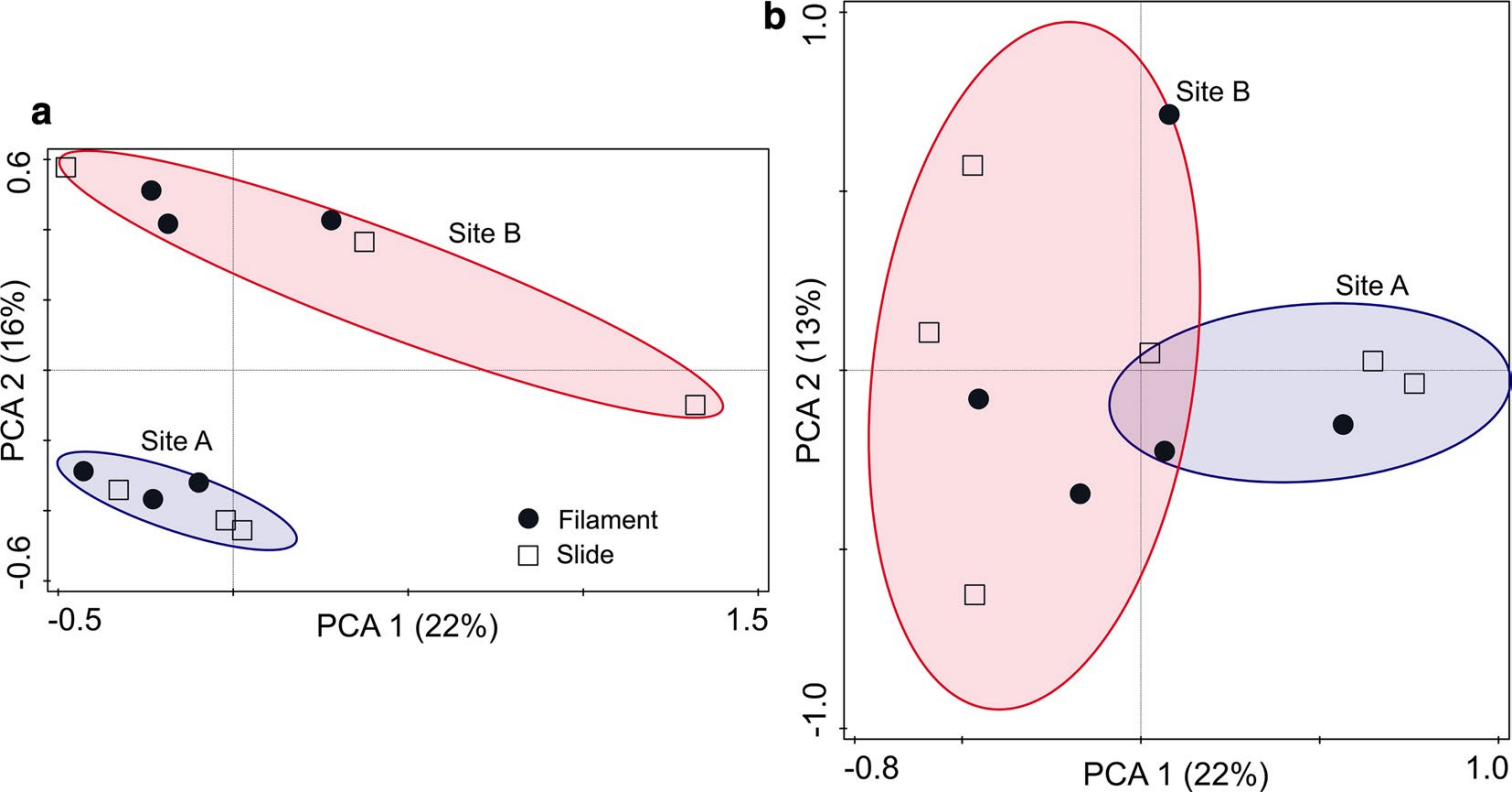
Principal component analysis (PCA) showing bacterial assemblages of each substrate at different sampling events **a** 7-day and **b** 14-day (Site A = lentic, Site B = lotic)

The effects of sampling time, site and substrate type on microbial communities attached to artificial substrates were examined using PCA and ANOSIM analyses. Although one sample failed to sequence, it had little influence on the results. PCA analysis showed that bacterial communities were largely overlapping for the two substrates, regardless of the sampling time. In contrast, Site A bacterial communities were distinct from the bacterial communities at Site B. Throughout the 14-day time course, the PCA analysis illustrated that differences in bacterial community structures between the two sites were diminished (Fig. 6). ANOSIM analyses further showed significant differences in bacterial community structure both between sites and between sampling times (Table 3). No significant difference was detected between the two substrates.

**Table 3:** Analysis of similarity (ANOSIM) comparisons for spatial, temporal and substrate differences in bacterial communities

Comparison	Sample statistic R	p
7 days vs. 14 days	0.482**	< 0.001
Site A (lentic) vs. Site B (lotic)	0.186**	0.008
Filament vs. slide	− 0.042	0.784

* p < 0.05; ** p < 0.01

## Discussion

Identification of microbes attached to artificial substrates in a hypereutrophic urban river revealed a complex microbial community structure. The results indicated that the microbial communities were composed of 80,375 OTUs distributed among 47 phyla. Attached microbial communities were dominated by the phyla *Proteobacteria*, *Cyanobacteria*/*Chloroplast*, *Planctomycetes*, *Actinobacteria*, *Verrucomicrobia*, *Firmicutes* and *Bacteroidetes*, with generally little variation in relative abundance between the two substrate types (Fig. 3). Our results are consistent with those of previous studies showing that *Proteobacteria* was the most abundant phylogenetic group in most river studies (Bricheux et al. 2013; Carney et al. 2015; Kochling et al. 2017), and biofilms in eutrophic waters were dominated by *Cyanobacteria* (Danilov and Ekelund 2002).

Sufficiently illuminated aquatic biofilms containing mixed communities are actively involved in the ammonia-oxidizing process (Anderson 2016; Coci et al. 2008, 2010). Hence, this study focused on the composition of ammonia-oxidizing microorganisms due to the high ammonium concentrations found in this hypereutrophic urban river. These complex attached microbial communities contained three genera of ammonia-oxidizing bacteria (*Nitrosomonas*, *Nitrosospira* and *Nitrosococcus*) and two genera of ammonia-oxidizing archaea (*Nitrososphaera* and *Nitrosopumilus*), of which the genus *Nitrosomonas* was the dominant in each sample, similar to a study from 10 wastewater treatment systems by Gao et al. (2014). Interestingly, over time, the relative abundance of the genus *Nitrosomonas* increased sharply, regardless of the attachment substrate and sampling site (Fig. 4). Compared with the water column, solid surfaces were more densely populated with ammonia-oxidizing bacteria in aquatic environments (Coci et al. 2008; Matulewich and Finstein 1978). The reasons may be that the extracellular polymeric materials produced by the microbes during the formation of biofilms in aquatic environments provide benefits for ammonia-oxidizing bacteria, and also the surface-attached growth has been proposed to offer resilience against environmental constraints (Powell and Prosser 1992). Obviously, the significant increase in the relative abundance of ammonia-oxidizing bacteria was linked to their ability to adapt to surface-attached growth. These results are consistent with the idea that nitrogen transformation processes in small-river ecosystems are primarily associated with the attached microbial communities (Herrmann et al. 2011; Lock 1993).

Microbial communities within aquatic biofilms have been proposed as potential bioindicator for changes in water quality due to their rapid response to environmental conditions and their great diversity (Witt et al. 2011). Therefore, we focus on whether the attached microbial communities can identify human-health concerns in urban river water quality. Considering polluted river waters may contain a large variety of pathogenic microorganisms (Servais et al. 2007), such human health risks are assessed by enumerating fecal indicator bacteria and pathogens. In the present experiment, the results of high-throughput sequencing documented the presence of fecal indicator bacteria and pathogens in microbial communities attached to artificial substrates in the Wen-Rui Tang River, although the relative abundance of fecal indicator bacteria and pathogens decreased gradually with time (Fig. 5). Unexpectedly, the genus *Legionella*, which is one of the main causative agents of severe atypical pneumonias (Yanez et al. 2005), was found in each sample. Our results support the premise that the Wen-Rui Tang River receives appreciable inputs of untreated sewage, which poses a serious risk to humans and environmental health.

The microbial communities attached to artificial substrates in this hypereutrophic urban river showed variable responses to sampling time, site and substrate type. Araya et al. (2003) reported that microbial community structures (determined by denaturing gradient gel electrophoresis, DGGE) in biofilms from an urban river matured within 3–7 days of their formation and did not change appreciably over longer time periods. However, results of cell counts from our study documented significant differences in the number of microbes attached on artificial substrates between sampling days 7 and 14 (Fig. 2). The analysis of the species richness and diversity of the surface-attached communities showed a regular decrease from 7 to 14 day (Table 2), suggesting that there were further changes in the surface-attached microbial communities after 7 days. The ANOSIM also indicated that significant differences in attached microbial community structures existed between the two sampling times (Table 3). One possible reason for the difference between our results and those of Araya et al. (2003) may result from the method used for studying the microbial community. In contrast to DGGE analysis, high-throughput sequencing provides more information on microbial community structure (Lemos et al. 2011; Logares et al. 2012).

In addition to temporal heterogeneity, the intra-river heterogeneity (lentic vs. lotic) in attached microbial community structure was significant. Although PCA analysis showed that attached microbial communities from the two sites became more similar over time, the attached microbial communities were clearly separated between the two sites (Fig. 6). Moreover, significant differences in the number and community composition of attached microbes between the two study sites were examined (Fig. 2 and Table 3). There were no marked differences in nutrient status between the two sites separated by about 300 m (Table 1), but the two sites had different hydrodynamic conditions. This may be a primary reason for the dissimilarity in attached microbial communities between the two sites.

Generally, a substrate is necessary for the attachment and the development of attached microbes in biofilms within aquatic environments. The physicochemical property of substrate may influence the attached microbial community structure (Dang and Lovell 2000). The two substrates used in the present study were both plastics and had similar surface properties. No significant differences in attached microbial community composition were detected between the two substrates in our study (Fig. 2, Table 3). However, compared with the slide, the number of bacteria attached on the filament showed a marked increase with time at Site A (Fig. 2). This difference in attached microbial number might be attributed to differences in the space structure and specific surface area characteristics of the two substrates.

In conclusion, our results revealed that microbial communities attached on artificial substrates in a hypereutrophic urban river were composed of 80,375 OTUs that distributed among 47 phyla. *Proteobacteria* and *Cyanobacteria*/*Chloroplast* were the two dominant phyla, followed by *Planctomycetes*, *Actinobacteria*, *Verrucomicrobia*, *Firmicutes* and *Bacteroidetes*. Further analysis of taxonomic composition showed that ammonia-oxidizing microorganisms, fecal indicator bacteria and pathogens were enriched in attached microbial communities, especially the ammonia-oxidizing bacteria of the genus *Nitrosomonas*. Furthermore, our results demonstrated that there were significant temporal and intra-river heterogeneity in the attached microbial community structure, but no significant difference was detected between the two substrate types. These results support the hypothesis that ammonium oxidation is a major source of oxygen demand in these hypereutrophic waterways. Providing instream artificial substrates (e.g., roots of floating wetlands, suspended woody or plastic materials) to enhance ammonium oxidation to nitrate with subsequent denitrification in the anoxic sediments maybe a mechanism to promote attenuation of excess nitrogen in these hypereutrophic waterways.

## Abbreviations

ACE: abundance-based coverage estimator
ANOSIM: analysis of similarity
DO: dissolved oxygen
NH_4_^+^–N: ammonium
NO_2_^−^–N: nitrite
NO_3_^−^–N: nitrate
OTUs: operational taxonomic units
PCA: principal component analysis
PO_4_^3−^–P: orthophosphate
TN: total nitrogen
TOC: total organic carbon
TP: total phosphorus
